# A Tale of Two Grass Species: Temperature Affects the Symbiosis of a Mutualistic *Epichloë* Endophyte in Both Tall Fescue and Perennial Ryegrass

**DOI:** 10.3389/fpls.2020.00530

**Published:** 2020-05-08

**Authors:** Priscila P. Freitas, John G. Hampton, M. Phil. Rolston, Travis R. Glare, Poppy P. Miller, Stuart D. Card

**Affiliations:** ^1^Bio-Protection Research Centre, Lincoln University, Lincoln, New Zealand; ^2^Forage Science, AgResearch Limited, Lincoln Research Centre, Lincoln, New Zealand; ^3^The Foundation for Arable Research, Christchurch, New Zealand; ^4^Knowledge and Analytics, AgResearch Limited, Grasslands Research Centre, Palmerston North, New Zealand; ^5^Forage Science, AgResearch Limited, Grasslands Research Centre, Palmerston North, New Zealand

**Keywords:** alkaloid, cool-season grass, lolines, mycelial biomass, peramine, vertical transmission

## Abstract

Many cool-season grasses form permanent, mutualistic symbioses with asexual *Epichloë* endophytes. These fungal symbionts often perform a protective role within the association as many strains produce secondary metabolites that deter certain mammalian and invertebrate herbivores. Although initially a serious issue for agriculture, due to mammalian toxins that manifested in major animal health issues, selected strains that provide abiotic stress protection to plants with minimal ill effects to livestock are now commercialized and routinely used to enhance pasture performance in many farming systems. These fungal endophytes and their grass hosts have coevolved over millions of years, and it is now generally accepted that most taxonomic groupings of *Epichloë* are confined to forming compatible associations (i.e., symptomless associations) with related grass genera within a tribe. The most desired compounds associated with *Epichloë festucae* var. *lolii*, an endophyte species associated with perennial ryegrass, are peramine and epoxy-janthitrems. No other major secondary metabolites with invertebrate bioactivity have been identified within this association. However, other agriculturally beneficial compounds, such as lolines, have been discovered in related endophyte species that form associations with fescue grasses. A rationale therefore existed to develop novel grass-endophyte associations between loline-producing endophytes originally isolated from tall fescue with elite cultivars of perennial ryegrass to achieve a wider spectrum of insect bioactivity. A suitable loline-producing endophyte strain of *Epichloë* sp. FaTG-3 was selected and inoculated into perennial ryegrass. We hypothesed that endophyte transmission frequency, endophyte mycelial biomass and endophyte-derived alkaloid production would differ between the original tall fescue host and the artificial association. Consistent with our hypothesis, our data strongly suggest that plant species significantly affected the plant-endophyte association. This effect became more apparent for transmission frequency and endophyte biomass as the plants matured. Overall, the viable endophyte infection frequency was greater in the tall fescue host than in perennial ryegrass, at all sampling dates. Additionally, temperature was found to be a significant factor affecting endophyte transmission frequency, endophyte mycelial biomass and alkaloid production. Implications for the development of novel grass-endophyte associations are discussed.

## Introduction

Members of the sub-family Pooideae (family Poaceae) form permanent, symbiotic associations with fungal endophytes of the genus *Epichloë* (family Clavicipitaceae) and their asexual morphs, previously known as *Neotyphodium* (Leuchtmann et al., 2014). The grass host provides shelter and nutrients to the endophyte, while the plant benefits through increased tolerance to abiotic and biotic stresses (Malinowski and Belesky, 2000; Popay and Bonos, 2005). Asexual *Epichloë* species have lost the power of contagion, being exclusively vertically transmitted via host seed after colonization of inflorescences, flower and seed tissues (Zhang et al., 2017). In New Zealand (NZ) agriculture, the most economically important associations are those between selected “animal friendly” *Epichloë festucae* var. *lolii* strains and elite cultivars of perennial ryegrass (*Lolium perenne*) (Easton, 2007; Johnson et al., 2013), the dominant pasture species cultivated in NZ (Valentine and Kemp, 2007).

These fungal-grass associations were detrimental to NZ agriculture around the late 1970s and early 1980s as some natural associations produced a number of mammalian toxins (indole-diterpenes, e.g., lolitrem B, and ergot alkaloids, e.g., ergovaline) that manifested in major animal health issues including ryegrass staggers, a neurological disorder (di Menna et al., 2012). Since this era, a great deal of fundamental research was undertaken with respect to the endophyte’s biology, chemistry and genetic diversity (Johnson et al., 2013). This knowledge led a NZ government-owned institute, AgResearch Limited, to develop an endophyte bioprospecting pipeline that identifies, characterizes and selects agriculturally beneficial strains (those that produce bio-protective properties to the host while conferring notably low or no detriment to grazing livestock) that can be incorporated into elite grass cultivars and marketed for increased pasture persistence and productivity (Johnson et al., 2013; Card et al., 2014; Bonth et al., 2015; Johnson and Caradus, 2019).

One of the most challenging steps in developing *Epichloë* endophytes for commerce is the ability to transfer suitable fungal strains from their original wild grass host to elite grass cultivars (Easton, 2007; Johnson and Caradus, 2019). The strain designated as AR1 was one of the first endophytes to be commercialized in 2001 (Johnson et al., 2013; Johnson and Caradus, 2019) and by 2008 over 70% of the proprietary seed sold in NZ was infected with this agriculturally beneficial fungal strain (Caradus et al., 2013). AR1 primarily produces the alkaloid peramine, which is responsible for insect deterrence, particularly toward Argentine stem weevil (ASW) while expressing no animal toxicity (Rowan and Gaynor, 1986; Rowan et al., 1986; Fletcher, 1999). The next leap in endophyte discovery arrived with the advent of epoxy-janthitrems, a unique indole diterpene compound with a wider range of insect deterrence than peramine and in 2006, strain AR37, a producer of this class of alkaloid was released onto the NZ market (Caradus et al., 2013; Johnson et al., 2013; Hennessy et al., 2016; Johnson and Caradus, 2019). No other major secondary metabolites with invertebrate bioactivity have been identified within this fungal taxon. However, other agriculturally beneficial alkaloids, such as lolines, have been discovered in related species that pre-dominantly form associations with tall fescue (*Festuca arundinacea*) and meadow fescue (*F. pratensis*) (Schardl et al., 2007). A rationale therefore existed to develop novel grass-endophyte associations between loline-producing endophytes originally isolated from tall fescue with elite cultivars of perennial ryegrass to achieve a wider spectrum of insect bioactivity than possible with ryegrass endophyte species (Easton, 2007; Easton et al., 2009).

A high frequency of viable endophyte infection in seed is desired by the seed industry for commercial endophyte products going to market, although this can be difficult to achieve with certain novel grass-endophyte associations (Rolston and Agee, 2007). Failure in vertical endophyte transmission has been documented for many endophyte-grass associations, including novel and wild-type associations, with no single factor responsible (Gundel et al., 2008). As well as genetic factors (Ju, 2011; Gagic et al., 2018), some environmental factors can contribute to incompatibility issues between these endophytic fungi and their grass hosts that may culminate by inhibiting the endophyte’s transmission pathway, with temperature suggested as one of the most important (Ju et al., 2006).

This study investigated the effects of different temperature regimes on the vertical transmission of an *Epichloë* endophyte strain within tall fescue (its original host species) and perennial ryegrass (a novel host species) and further analyzed endophyte infected plants with respect to their mycelial biomass and production of insect deterrent alkaloids. We hypothesed that endophyte transmission frequency, endophyte mycelial biomass and endophyte-derived alkaloid production would differ between the original tall fescue and the novel, or artificial, association developed with perennial ryegrass.

## Materials and Methods

Two grass lines were used in this study, namely T9886, a tall fescue line, cv. Grasslands Flecha (a summer dormant Mediterranean-type cultivar) and KLp903, a tetraploid perennial ryegrass line, derived from crosses of the cultivars Banquet, Banquet II and Bealey. Both seed lines were infected with the same strain of fungal endophyte, *Epichloë* sp. FaTG-3, strain AR501, previously designated TF16 (Christensen et al., 1993). AR501 produces peramine and loline alkaloids but none of the ergot or indole diterpene alkaloids linked to animal toxicosis when associated with its original native grass host or within novel (or artificial) associations with tall fescue or perennial ryegrass. The lack of animal toxins is due to the absence of key genes in both alkaloid pathways. The seeds of the tall fescue line were harvested in 2012 and subsequently stored at near optimal storage conditions for endophyte, 0°C with 30% relative humidity (Rolston et al., 1986; Rolston et al., 1991) in the Margot Forde Germplasm Center, New Zealand’s national gene-bank of grassland plants. Seed from the perennial ryegrass line was harvested in 2011 and stored by PGG Wrightson Seeds Ltd. under the same conditions as for the tall fescue line. The viable endophyte infection frequencies of each grass line were determined before the experiment commenced by assessing ∼96 seedlings per line using an established tissue-immunoblot technique (Simpson et al., 2012).

**FIGURE 1:**
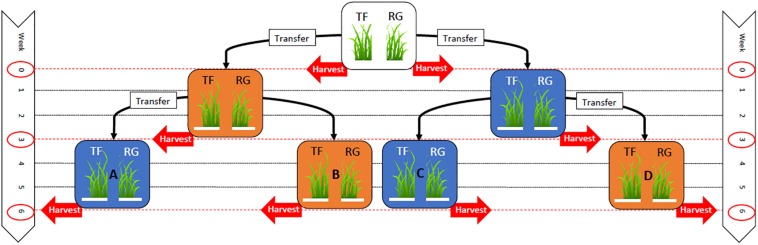
Experimental layout indicating the timeline where 28 trays of tall fescue (TF) and 28 trays of perennial ryegrass (RG) plants associated with the endophyte *Epichloë* sp. FaTG-3, strain AR501, were originally grown within a glasshouse until they were two weeks old (top white box). At week 0, eight trays (four trays of TF and four trays of RG) were removed for harvesting, while the remaining 48 trays (24 trays of TF and 24 trays of RG) were transferred to either a warm (25/16°C: day/night) growth chamber, symbolized with an orange box, or a cool (12/6°C: day/night) chamber, symbolized with a blue box, for three weeks. At week 3, 16 trays (four trays of TF from the cool environment, four trays of RG from the cool environment, four trays of TF from the warm environment and four trays of RG from the warm environment) were removed for harvesting, while the remaining 32 trays were either left in the same environment or transferred to a different environment (either a warm or cool temperature regime depending on the environmental regime they had previously been subjected to) for a further three weeks. At week 6, the remaining 32 trays of TF and RG from all environmental regimes were removed for harvesting.

Seeds from each seed line were sown in separate 24-cell plastic trays (196 cm^3^ per cell) containing seedling mix (120 L Southland peat, 80 L pumice) with the following fertilizer additions per cubic meter: 4 kg Osmocote^®^ exact mini (16% N, 3.5% P, 9.1% K), 8 kg dolomite lime and 2 kg Hydroflo^®^ (granular wetting agent manufactured by Everris Australia Ltd.). Two seeds were sown in each cell at a depth of 1 cm. The experiment was set up in October 2013 using a randomized block design with four blocks and 14 trays in each block (56 trays in total, 28 trays of tall fescue and 28 trays of perennial ryegrass). All the trays were initially placed in a heated glasshouse (∼20°C) and watered as required until the seedlings had emerged. Two weeks after sowing, seedlings were thinned to one seedling per cell by hand, and the trays randomly assigned to treatment groups consisting of four controlled temperature regimes (A–D, see Figure 1).

At week 0, at the commencement of the experiment, two trays from each block (four trays containing tall fescue plants and four trays containing perennial ryegrass plants), eight trays in total, were randomly selected and six plants per tray were randomly selected and harvested. This involved removing seedlings from their cell trays and separating the foliar tissues (pseudostem and leaves) from root tissues with the aid of a scalpel. The foliar tissues were bulked and freeze dried using a bench top freeze dryer (MicroModulyo, Thermo Savant^TM^, United States) and ground using a coffee grinder (Breville Group Ltd., China) before being stored at –20°C. The root samples were discarded. The biomass of endophyte mycelia per plant was then determined using an enzyme-linked immunosorbent assay (ELISA) developed by AgResearch (Faville et al., 2015). The remaining 48 seedling trays were split into two groups, of an equal number of trays and grass species, and transferred from the glasshouse to two walk-in growth chambers (PGV36, Conviron^®^, Canada) with a photoperiod length of 16 h light/8 h dark. One chamber with a temperature regime set at 25/16°C: day/night (termed from now on as warm) and the other chamber set at 12/6°C: day/night (termed from now on as cool). The light intensity was 244 and 214 μmol m^–2^ s^–1^ for the cool and warm growth chambers, respectively.

At week three, eight trays from each of the two temperature regimes (warm and cool), 16 trays in total, were randomly selected and six plants per tray were harvested as described previously. Additionally, the viable endophyte infection frequency of these plants was assessed as described earlier. At this time, eight of the sixteen remaining trays from the warm temperature regime were placed into the cool temperature regime, and eight of the sixteen remaining trays from the cool temperature regime were placed into the warm temperature regime (Figure 1). Plants were left to grow for a further three weeks before six plants per tray were again randomly harvested and assessed for viable endophyte (week 6 harvest). Plants (including tillers previously assessed by TPIB) were freeze dried, ground and endophyte mycelial biomass plus the concentration of agriculturally beneficial grass-endophyte derived alkaloids, peramine and lolines, determined by ELISA. Samples were analyzed for peramine and loline alkaloids using ELISAs developed by AgResearch (Briggs et al., 2017). The plate coating conjugate, and the polyclonal sheep anti-peramine antibody were originally described by Garthwaite et al. (1994) but the immunoassay was reformatted and all other reagents, buffers and the protocol were replaced (Briggs, pers. comm.).

At all harvest dates (week 0, 3, and 6) trays of tall fescue and perennial ryegrass were removed from the growth chambers and six plants per tray randomly selected and harvested. At week 0, a total of 48 plants were selected from eight trays (4 trays of tall fescue and 4 trays of perennial ryegrass) and assessed for mycelial biomass. Plants at this stage were too small to be assessed for the presence of viable endophyte by the tissue print immunoblot technique. At week 3, a total of 96 plants were selected from 16 trays (8 trays of tall fescue and 8 trays of perennial ryegrass) and assessed for mycelial biomass and their endophyte infection status. At week 6, a total of 192 plants were selected from 32 trays (16 trays of tall fescue and 16 trays of perennial ryegrass) and assessed for mycelial biomass, their endophyte infection status and alkaloid concentration as described previously. In total, 336 plants were harvested from the three harvest dates.

Statistical analyses for the endophyte frequency data was performed using R (R Core Team, 2019), whilst the mycelial biomass data was analyzed using GenStat for Windows (18th Edition, VSN International, Hemel Hempstead, United Kingdom). A binomial generalized linear mixed model (GLMM) was used to model endophyte infection frequencies using lme4 (Bates et al., 2015), where block was treated as a random effect. Pairwise comparisons for the GLMM used Tukey’s *P*-value adjustment for multiple comparisons using the emmeans R package (Lenth et al., 2019) with statistical significance defined as *P* < 0.05. Analysis of variance (ANOVA) and Fisher’s protected test of least significant difference (LSD; *P* < 0.05) were performed to compare treatment effects for the mycelial biomass and alkaloid concentration data. Mycelial biomass and alkaloid (peramine and lolines) concentrations were natural log transformed prior to analysis to stabilize the variance. Loline (*N*-acetyl loline and *N*-formyl loline) and peramine concentrations in both perennial ryegrass and tall fescue hosts were each regressed against endophyte mycelial biomass to investigate potential relationships. Analysis of covariance, which fitted parallel lines through the scatter plots (one point per treatment) for the two grass hosts, was used to correlate alkaloid and endophyte biomass within hosts, after adjusting for differences in overall mean values between hosts.

## Results

### Endophyte Infection Frequency

The initial viable endophyte infection frequency of AR501 was 99 and 87% for the tall fescue and perennial ryegrass lines, respectively. At week 0 (the first harvest date), the endophyte infection frequency was not determined as the plants were too young, and therefore too fine, for assessment using the tissue print-immunoblot technique. At week 3 (the second harvest date), there was no variation in the tall fescue data. Therefore, the analysis only compared the data from the ryegrass datasets (cool vs. warm) and observed means rather than fitted means displayed in Table 1. The viable endophyte infection frequency of AR501 was significantly lower (32%) in the perennial ryegrass line exposed to the warm temperature regime compared to the ryegrass line exposed to the cool temperature regime (84%) (Tables 1, 2). Additionally, there was a slight trend indicating a higher endophyte infection frequency of AR501 in tall fescue than perennial ryegrass (Table 1).

**TABLE 1:** Fitted mean % of viable endophyte infection (95% CI) and mean mycelial biomass (± SD) of *Epichloë* sp. FaTG-3 strain AR501 within perennial ryegrass and tall fescue host backgrounds after plants were incubated for fixed periods at selected temperature regimes.

	**Fitted mean % of viable**		**Mean mycelial biomass**	
	**endophyte infection (95% CI)**		**(mg/g ± SD)**	
**Temperature regime (day/night)**	**Perennial ryegrass**	**Tall fescue**	**Perennial ryegrass**	**Tall fescue**
**Initial seed infection frequency**				
∼20°C constant	87*	99*	n/a	n/a
**Week 0**				
∼20°C constant	nt	nt	1.24 (± 0.56) a	2.22 (± 2.66) a
**Week 3**				
12/6°C (cool)	84 (52, 99) a	100*	1.07 (± 0.11) a	2.27 (± 0.54) a
25/16°C (warm)	32 (4, 73) b	100*	0.57 (± 0.24) a	1.45 (± 1.29) a
**Week 6**				
12/6°C (cool)	75 (54, 88) a	96 (76, 99) a	2.72 (± 0.46) b	1.92 (± 0.41) ab
25/16°C (warm)	75 (54, 88) a	92 (72, 98) a	2.73 (± 1.07) b	7.29 (± 1.80) d
25/16°C transferred to 12/6°C (warm to cool)	58 (38, 76) a	87 (68, 96) a	0.81 (± 0.42) a	*1.93 (± 0.64)* ab
12/6°C transferred to 25/16°C (cool to warm)	75 (54, 88) a	92 (72, 98) a	5.18 (± 1.02) c	10.82 (± 1.70) e

Means within a sampling period (e.g., week 6) followed by the same letter are not significantly (P > 0.05) different as determined by Tukey’s HSD test for the week 6 endophyte infection data or Fisher’s protected LSD test for mycelial concentration data.*No variation in the data, therefore observed means are displayed rather than fitted means, n/a is not applicable, nt is not determined as the tillers were too young, and therefore too fine, to be assessed by the tissue print-immunoblot technique.

**TABLE 2:** *P*-values for the effects of plant species (perennial ryegrass and tall fescue), temperature (cool and warm for weeks 0-3, and cool, warm, transfer from cool to warm and transfer from warm to cool for week 6) on *Epichloë* sp. FaTG-3 strain AR501 endophyte infection frequencies (%) and mycelial biomass (mg/g).

**Source of variation**	***P*-value**		
	**Week 0**	**Week 3**	**Week 6**
**Viable endophyte infection frequency**			
Plant species	–	–	**< 0.001**
Temperature	–	**0.002**	0.385
Plant species × Temperature	–	–	0.950
**Biomass of endophyte mycelia**			
Plant species	0.495	**0.004**	**< 0.001**
Temperature	–	**0.005**	**< 0.001**
Plant species × Temperature	–	0.973	**0.001**

*P-values for endophyte infection frequency data were generated via Type II Wald Chi-square tests, whilst P-values for endophyte mycelia data were generated by Fisher’s protected LSD test (P < 0.05). Values in bold are statistically significant (P < 0.05), – no analysis possible.*

At week 6 (the third harvest date), there were no overall significant interactions between plant species and temperature, although there was a slight trend indicating that plants transferred from the warm to the cold temperature regime showed a lower viable endophyte infection frequency, regardless of host plant species (Tables 1, 2). There was also a significant (*P* < 0.001) effect of plant species, i.e., there was a higher viable endophyte infection frequency in tall fescue than perennial ryegrass (Table 2).

### Biomass of Endophyte Mycelia

At week 0 (the first harvest date), the mycelial biomass of AR501 did not differ significantly between the tall fescue (2.22 mg/g) and perennial ryegrass (1.24 mg/g) host populations grown at 20°C (Tables 1, 2). At week 3 (the second harvest date), there was no overall significant interaction between treatments (Tables 1, 2), although there was a significant (*P* = 0.004) effect of host plant species, i.e., there was a greater amount of endophyte mycelia detected in tall fescue than perennial ryegrass (Tables 1, 2) and a significant (*P* = 0.005) effect of temperature on the biomass of mycelia within endophyte-infected plants. At week 6 (the third harvest date), there was a highly significant interaction between treatments (*P* = 0.001) indicating that plant species and temperature influenced the biomass of endophyte mycelia (Tables 1, 2). Within the perennial ryegrass treatments, the lowest amount of endophyte mycelia (0.81 mg/g) was detected in plants transferred from the warm to the cool temperature regime (Table 1). For tall fescue, the lowest amount of mycelia was also detected in the plants transferred from the warm to the cool temperature regime (1.93 mg/g) and for plants kept at the cool temperature regime (1.92) for the entire experiment (Table 1). The greatest biomass of endophyte mycelia was detected in plants transferred from the cool to the warm temperature regime, regardless of host plant species, with a mean of 5.18 mg/g detected in perennial ryegrass plants and 10.82 mg/g detected in tall fescue (Table 1).

### Concentration of Insect Deterrent Alkaloids

At week 6 (the third harvest date), there were no overall significant interactions among treatments (Tables 3, 4), although there were highly significant (*P* < 0.001) effects with regards to temperature for both peramine and loline concentrations. The lowest concentrations of peramine were recorded for plants transferred from the warm to cool temperature regime and the highest concentrations recorded for plants transferred from the cool to warm temperature regime, irrespective of plant species (Table 3). For lolines, the greatest concentrations were again recorded for plants transferred from the cool to warm temperature regime (Table 3). Additionally, there was a highly significant (*P* < 0.001) effect of plant species on the production of loline alkaloids indicating that more lolines were produced by tall fescue plants infected with AR501 than perennial ryegrass (Tables 3, 4). There was a near significant (*P* = 0.052) effect with respect to plant species on the production of peramine (Tables 3, 4).

**TABLE 3:** Mean concentration of peramine and total loline alkaloids at the week 6 harvest in perennial ryegrass and tall fescue plants, infected with the endophyte *Epichloë* sp. FaTG-3, strain AR501.

	**Mean concentration**		**Mean concentration of**	
	**of peramine**		**total loline alkaloids**	
	**(μg/g ± SD)**		**(μg/g ± SD)**	
**Temperature regime (day/night)**	**Perennial ryegrass**	**Tall fescue**	**Perennial ryegrass**	**Tall fescue**
12/6°C (cool)	5.62 (± 2.99) cde	4.22 (± 1.56) bcd	1.50 (± 0.00) a	14.78 (± 8.08) b
25/16°C (warm)	4.26 (± 3.24) bc	11.02 (± 5.40) ef	7.30 (± 7.68) a	89.53 (± 74.73) c
25/16°C and transferred to 12/6°C (warm to cool)	1.53 (± 1.47) a	2.92 (± 1.78) b	1.50 (± 0.00) a	27.00 (± 19.26) b
12/6°C and transferred to 25/16°C (cool to warm)	12.03 (± 6.25) ef	17.28 (± 3.89) f	26.05 (± 18.36) bc	171.30 (± 79.30) d

*Means associated with the same alkaloid followed by the same letter are not significantly different as determined by ANOVA and Fisher’s protected LSD test (P > 0.05).*

**TABLE 4:** *P*-values for the effects of Plant Species (perennial ryegrass and tall fescue), Temperature (cool, warm, transfer from cool to warm and transfer from warm to cool) and their interaction with respect to the concentration of peramine and loline alkaloids (μg/g) associated with the endophyte, *Epichloë* sp. FaTG-3, strain AR501.

	***P*-value**	
**Source of variation**	**Peramine**	**Lolines**
Plant species	0.052	**<0.001**
Temperature	**<0.001**	**<0.001**
Plant species × Temperature	0.317	0.966

*P-values were generated by Fisher’s protected LSD test (P < 0.05). Values in bold are statistically significant (P < 0.05).*

### Correlation Between Mycelial Biomass and Alkaloid Concentration

**FIGURE 2:**
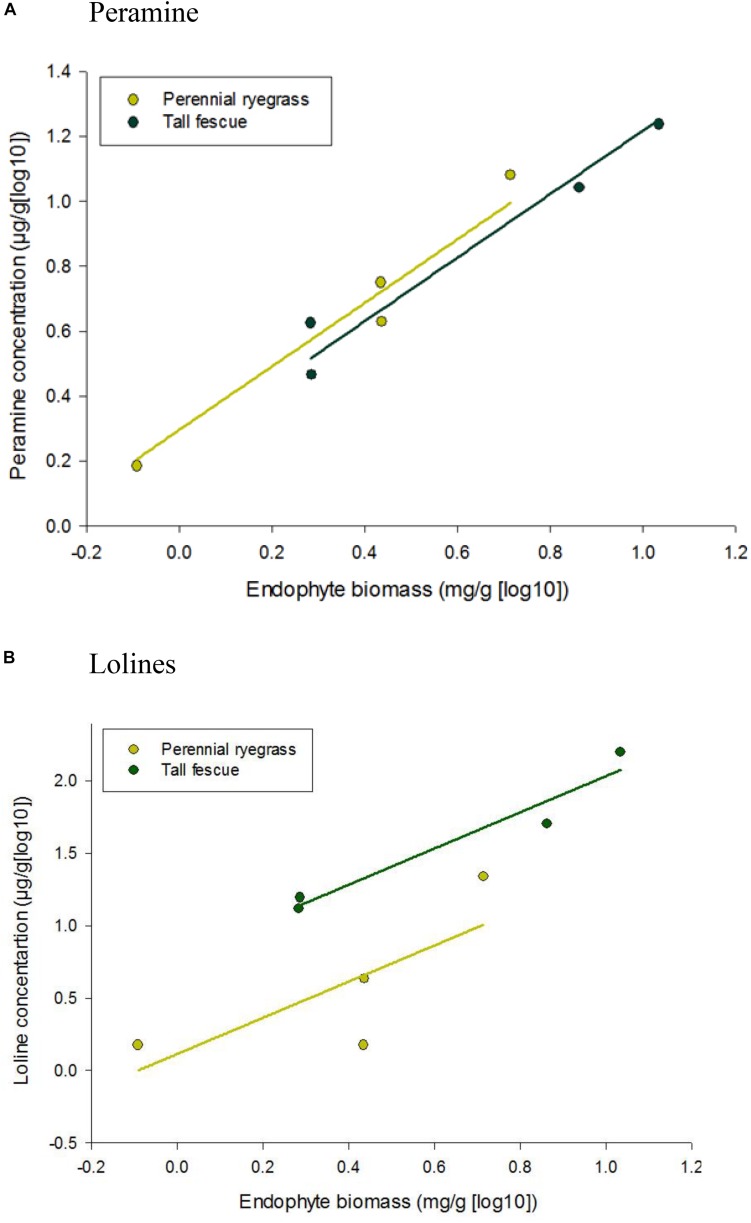
Correlation between alkaloid concentration, **(A)** peramine and **(B)** lolines, and endophyte biomass of *Epichloë* sp. FaTG-3 strain AR501 in perennial ryegrass and tall fescue plant hosts. Equations for fitted curves were **(A)** perennial ryegrass, y = 0.979x + 0.2955 and tall fescue, y = 0.979x + 0.2391 and **(B)** perennial ryegrass, y = 1.25x + 0.1143 and tall fescue, y = 1.25x + 0.7832. The parallel lines were fitted using analysis of covariance. Note that the scale on the y axis differs for each alkaloid.

Peramine and loline alkaloid concentrations increased when endophyte biomass increased (Figure 2), with the tall fescue endophyte association producing greater amounts of both alkaloids [1.04 and 1.70 mg/g (log10) of peramine and loline alkaloids, respectively] when endophyte biomass reached its maximum levels. The common slope of the parallel regression lines was significantly (*P* = 0.012 and *P* < 0.001) positive for both loline and peramine alkaloids, respectively.

## Discussion

We hypothesed that the endophyte transmission frequency, endophyte mycelial biomass and endophyte-derived alkaloid production would differ between associations formed between endophyte strain AR501 and the original host species, tall fescue, and the novel association developed with perennial ryegrass. Consistent with our hypothesis our data strongly suggests that plant species significantly affects the plant-endophyte association. This effect became more apparent for transmission frequency and endophyte biomass as the grass plants matured. Overall, the viable endophyte infection frequency was greater in the tall fescue host than in perennial ryegrass, at all sampling dates. However, as the endophyte infection frequency was higher in the original seed line of tall fescue compared to perennial ryegrass, and as asexual *Epichloë* cannot naturally infect endophyte-free plants due to their lack of horizontal transmission (Zhang et al., 2017), it was not surprising that this trend continued at all subsequent sampling dates. What was surprising was the effect of temperature at the second sampling date, which negatively impacted the endophyte infection status of perennial ryegrass but not tall fescue. However, the subsequent assessment of the endophyte infection frequencies at the final sampling date, showed that the perennial ryegrass plants that were exposed to the warm temperature regime for the first three weeks recorded a higher infection frequency. A possible explanation for this anomaly is that these plants had a mixed infection status (plants possessed both endophyte infected and endophyte-free tillers) and this ratio changed between the two sampling dates. An alternative, and possibly a more plausible explanation, is that as a low mycelial biomass was also detected in plants exposed to the warm temperature regime at the second sampling date, this could have resulted in false-negative blots developing on the nitrocellulose membrane used to determine the viable endophyte infection status of grass tillers (Gwinn et al., 1991). Ju et al. (2006), also used an immunoblot technique when studying tall fescue cv. Jesup infected with the MaxQ endophyte and noted a low endophyte infection frequency in winter compared to summer and this correlated to a low endophyte biomass within individual plants.

In addition to plant host species, temperature was also found to be a significant factor affecting the endophyte transmission frequency, endophyte mycelial biomass and endophyte-derived alkaloid production of strain AR501. At week 6, the biomass of AR501 mycelia was highest for both plant species when plants were grown in the cool temperature regime for three weeks and then transferred to the warm temperature regime. The biomass of endophyte mycelia within tall fescue was, however, almost twice as that detected in the perennial ryegrass association. When this situation was reversed (i.e., plants were transferred from the warm to the cool temperature regime), both grass hosts also had significantly less mycelium than if they were subjected to a constant warm temperature regime. Although the concentration of peramine and loline alkaloids were generally higher in the tall fescue host, trends for both plant species remained similar and constant. Peramine significantly increased when plants of both grass species were transferred from the cool to the warm temperature regime, compared to plants grown at a constant cool temperature. This resulted in a 2-fold increase for perennial ryegrass and a 4-fold increase for tall fescue. This trend was more dramatic for loline alkaloids with a 17-fold increase for perennial ryegrass and a 12-fold increase for tall fescue. The opposite trend was detected when plants of both species were transferred from the warm to the cool regime.

Other researchers have also recorded strong impacts of temperature on endophyte mycelial biomass and endophyte-derived alkaloids (Reinholz and Paul, 2000; Ryan et al., 2015; Fuchs et al., 2017). In our study the concentration of both peramine and loline alkaloids was highly correlated to the biomass of endophyte mycelia and this has been documented previously in many grass-endophyte associations and as mycelial biomass increases so do the concentration of these alkaloids (Bush et al., 1993; Keogh et al., 1996; Easton et al., 2002; Rasmussen et al., 2007; Ryan et al., 2015). Endophyte-derived alkaloids are independently regulated and are controlled by both plant and endophyte genotype (Roylance et al., 1994). These secondary metabolites are a product of the grass-endophyte association and although there are reports of *in vitro* production of some compounds from axenically cultured endophyte strains (Blankenship et al., 2001), the concentrations are minimal to those produced *in planta* (TePaske et al., 1993; Adhikari et al., 2016). Fuchs et al. (2017) showed that climatic conditions in spring and summer enhanced endophyte growth and alkaloid production (peramine, ergovaline and lolitrem B) in perennial ryegrass infected with *E. festucae* var. *lolii*. Additionally, Justus et al. (1997) showed that the highest concentrations of loline alkaloids were found in early spring and summer in meadow fescue infected with the endophyte *E. uncinata*. All these factors are ecologically linked, showing that alkaloids that protect against herbivory are produced when the threat of herbivory is at its highest. Endophytes are now known to play a major role in the structure of grassland communities and tropic interactions are likely to have significant consequences on the entire ecosystem (Saikkonen et al., 2016). The growth of *Epichloë* hyphae is synchronized with that of the plant with the endophyte behaving more like a plant tissue than a separate organism (Christensen et al., 2008). As both the fungus and plant have co-evolved over a period of 40 million years (Schardl et al., 2008), it is not unexpected that the association has developed ways to adapt to environmental cues in order to protect the plant from herbivory via the production of alkaloids. As these compounds are energy-rich, the association has also adapted ways to efficiently manage the production of these alkaloids.

High concentrations of endophyte-derived alkaloids are also found in seed, sometimes at higher levels than in vegetative plants, and probably evolved to reduce the probability of predation by granivores such as insects, small rodents and birds (Madej and Clay, 1991; Knoch et al., 1993; Finch et al., 2016; Pennell et al., 2017). Even seeds containing endophyte that has died can retain high alkaloid concentrations, which can protect seed from predation (Stewart, 1985; Ball et al., 1993). With the onset of seed germination, these compounds are subsequently translocated into seedlings to provide protection to the developing plant, although there is a period of vulnerability toward certain herbivores, such as ASW, up until the seedlings are six weeks old as these alkaloids are diluted within the increasing plant biomass (Ruppert et al., 2017). At six weeks it is then speculated that the endophyte becomes fully metabolically active and production of endophyte-derived alkaloid production can then commence. In a mature plant, alkaloid levels are generally highest in young leaves, stems and panicles (Saikkonen et al., 2013). Concentrations and the ratio of the different loline alkaloids are also dependent upon endophyte and host genotype (Ball and Tapper, 1999). Unfortunately assessing the different types of loline produced by AR501 within tall fescue and perennial ryegrass was outside the scope of this study. This would be advantageous information to gain from future research as the three loline alkaloids, *N*-formyl loline (NFL), *N*-acetyl loline (NAL), and *N*-acetyl norloline (NANL), have differing bioactive properties. Some tall fescue associations lack NFL and NAL and some lack lolines completely (Ball and Tapper, 1999). Concentrations of NFL, NAL, and NANL were generally higher in meadow fescue than tall fescue when infected with the same endophyte genotype. The study by Ball and Tapper (1999) also showed that mycelial biomass and production of the alkaloids peramine and lolines were heavily influenced by host plant species with greater amounts of mycelia and secondary metabolites produced in tall fescue. Additionally, recent research has uncovered greater diversity in the pyrrolopyrazines (i.e., peramine) than previously thought (Berry et al., 2019) and future investigations need to account for the potential derivations produced by these fungal endophytes.

*Epichloë* strain AR501, an FaTG-3-type endophyte, was originally isolated from a Mediterranean-type tall fescue plant growing in southern Spain and, as with other strains of this grouping, is an asexual hybrid formed between *Epichloë baconii* and *E. typhina* (Moon et al., 1999). Endophyte strains from this taxonomic grouping are known for their production of peramine and loline alkaloids but produce none of the metabolites linked to animal toxicity (i.e., ergovaline). As only one single AR501-infected plant of the original wild accession was present within the AgResearch parent plant collection, an alternative host was required for experimental purposes. Cultivar Grasslands Flecha, a summer dormant, Mediterranean cultivar of tall fescue, with a similar genetic background to the original host, was chosen as the host grass for AR501 alongside perennial ryegrass (line KLp903) for experimentation. Flecha was selected from germplasm native to the Mediterranean region and can exhibit less summer activity and more winter growth than cultivars developed from germplasm indigenous to central and northern Europe (Norton et al., 2006; He et al., 2017). Therefore, we have assumed that the endophyte biomass, chemical profile and colonization behavior of AR501 would be similar within Flecha as with the original wild *Festuca* host.

Christensen et al. (1993) were the first to investigate and document the compatibility (or lack of) within novel or artificially developed *Epichloë*-grass associations. After creating novel associations using endophyte strains from six recognizable taxonomic groupings and inoculating them into the apical meristem of three grass species, symptoms of incompatibility were observed. These included stunted tillers and necrosis of the apical meristem. Koga et al. (1993) documented less severe symptoms within incompatible novel associations created with perennial ryegrass and *E. coenophiala*. These symptoms, however, only affected the endophyte rather than the plant, and manifested as distorted, collapsed and dead hyphae. *Epichloë* fungal endophytes and their grass hosts have coevolved, and it is now generally accepted that endophyte taxonomic groupings, including those from tall fescue, are not host-specific but are confined to forming compatible associations (i.e., symptomless associations) with related grass genera within a tribe (Schardl and Phillips, 1997; Schardl et al., 1997). Based on chloroplast genome analysis, both tall fescue and perennial ryegrass are classified within chloroplast group 2 of the subtribe Lollinae, with rationale for aligning *Festuca* subg. *schedonorus*, the broad-leafed fescue species with the inclusion of tall fescue, within the genus Lolium (Darbyshire, 1993). The severe symptoms (described by Koga et al., 1993; Christensen, 1995) from incompatible endophyte-grass associations were not observed with the novel associations used for experimental purposes described in our study. We speculate that the close phylogenetical proximity of the two grass species is an explanation for this. Whether low endophyte transmission frequencies and low mycelial biomass within plants is a symptom of grass-endophyte incompatibility is an area of contention as low frequencies of endophyte can be found throughout many natural grass stands (Leyronas and Raynal, 2001).

Tall fescue is a long-lived, perennial, bunchgrass indigenous to Europe. Tall fescue can form mutualistic associations with asexual *Epichloë* endophyte species, all of which are interspecific hybrids derived from one or two parasexual hybridization events. These events likely occurred when an endophyte-infected plant was colonized by *Epichloë* ascospores of a different species, subsequently leading to anastomosis followed by karyogamy (Tsai et al., 1994; Schardl and Craven, 2003). Tall fescue typically associates with three taxonomic groupings of *Epichloë*, as defined by isozyme, alkaloid and morphological characteristics, namely *Festuca arundinacea* taxonomic group 1 (FaTG-1) = *E. coenophiala*, FaTG-2 and FaTG-3, with the latter two groupings yet to receive Linnaean names (Christensen et al., 1993; Christensen, 1995; Leuchtmann et al., 2014). These associations are believed to have coevolved over thousands of years with *E. coenophiala* possibly predating its tall fescue host, being identified within the ancestral tetraploid grass *Festuca arundinaceae* spp. *fenas* (Schardl and Craven, 2003; Vazquez de Aldana et al., 2003). Tall fescue is an outbreeding, allohexaploid species and is more accurately described as a species complex (Hand et al., 2010; Ekanayake et al., 2012) with three distinct morphotypes described; a Mediterranean morphotype indigenous to North Africa, a Continental morphotype indigenous to Northern Europe and a rhizomatous morphotype indigenous to the Iberian Peninsula (Dierking et al., 2012). *E. coenophiala* associates with the Continental morphotype while FaTG-2 and FaTG-3 associate closely with the Mediterranean morphotype. Little information is available on the endophyte associations formed with the rhizomatous grass morphotype. However, this morphotype shares the same progenitors as the Continental type, namely *F. pratensis* and *F. arundinacea* var. *glaucescens* (Hand et al., 2010) and therefore may naturally associate more closely with *E. coenophiala* than the other taxonomic groups.

Perennial ryegrass is native to southern Europe, the Middle East, North Africa and eastwards to central Asia. The low growing, tufted, hairless grass is now regarded as an important forage species in many countries around the world including Australia, New Zealand, North America and South Africa. This grass species naturally associates with two taxonomic groupings of mutualistic asexual *Epichloë* endophyte, namely *Lolium perenne* taxonomic grouping 1 (LpTG-1 = *Epichloë festucae* var. *lolii*) and LpTG-2 (= *Epichloë hybrida*). Perennial ryegrass does not respond well to hot temperatures during its establishment stage (Kemp et al., 1999) and we speculate that the low biomass of AR501 mycelia detected at the second harvest date (week 3) within this grass host, as compared to tall fescue, was due to the plant exhibiting stress under the warmer temperature regime. By the third harvest date (week 6) the perennial ryegrass-endophyte association was less susceptible to this environmental stress as indicative of a similar biomass of hyphae being detected in plants exposed to a constant warm or a constant cool temperature regime. In contrast, at week 6, the biomass of endophyte mycelia within the tall fescue host was lowest at the constant cool temperature regime and when plants were transferred after three weeks from the warm to the cool temperature regime. This was highly significant, with mycelial biomass 80% less in plants exposed to both these temperature regimes compared to plants transferred after three weeks from a cool to a warm temperature regime.

Christensen et al. (2008) established that *Epichloë* species colonize their grass hosts by a unique mechanism termed intercalary hyphal extension and not by the general model of hyphal tip growth. However, there is still substantial variation within the *in-planta* colonization patterns linked to different groups of these *Epichloë* species. For example, *Epichloë occultans*, associated with annual ryegrasses, are found as a dense mycelial mass located at the base of the leaf sheath while the hyphae of most *Epichloë* species are found throughout the leaf sheath, aligned to the leaf axis and are seldom branched (Christensen et al., 2002). Leaf blade colonization can differ between *Epichloë* species, host, genotype, novel and natural associations and with plant age (Christensen et al., 2002). Tall fescue associations differ in their distribution of *Epichloë* hyphae with continental-type associations exhibiting poor colonization of their leaf blades compared to Mediterranean associations where hyphae can be readily observed in blades and sheaths (Christensen and Voisey, 2006; Takach et al., 2012). It is assumed that the dense ligular zone found between the leaf sheath and blade is a physical barrier to the advancement of *Epichloë* hyphae. As grass leaves age, hyphae increase in diameter but not in frequency (Christensen and Voisey, 2006) and this could therefore have implications for the accumulation of mycelial biomass. Further research with AR501 would aim to determine if the distribution of hyphae differed between the perennial ryegrass and tall fescue hosts and if this had any significant effect on mycelial biomass and/or production of secondary metabolites.

Host and endophyte genotype, plant age, nutrition and environmental conditions (such as air and soil temperature, day length, solar radiation, and precipitation) are critically important factors in the production of endophyte-derived secondary metabolites (Bush et al., 1993; Reinholz and Paul, 2000; Krauss et al., 2007; Fuchs et al., 2017). Concentrations of certain alkaloids, such as lolines, can also be stimulated *in-planta* after wounding, e.g., after insect attack (Schardl et al., 2007), showing that the grass-endophyte association is able to respond not only to biotic cues but also abiotic ones. Herbivory is one of the most important threats for plants, impacting net primary productivity in natural ecosystems and causing important economic losses in agriculture (Bastias et al., 2017). It is therefore logical that these grass plants would have devised complex mechanisms to enhance their fitness by associating with alkaloid producing Clavicipitaceous endophytic fungi.

## Data Availability Statement

The datasets generated for this study are available on request to the corresponding author.

## Author Contributions

PF carried out this research as a part of her Doctor of Philosophy (Research) and performed the experiments. JH, MR, TG, and SC were co-supervisors. PF, JH, MR, TG, and SC contributed conception and design of the study. PM, PF, and SC performed the statistical analysis. PF and SC wrote the first draft of the manuscript. All authors contributed to manuscript revision, read and approved the submitted version.

## Conflict of Interest

SC and PM were employed by the company AgResearch Limited. The remaining authors declare that the research was conducted in the absence of any commercial or financial relationships that could be construed as a potential conflict of interest. The authors declare that this study received funding from Grasslanz Technology Limited. The funder was not involved in the study design, collection, analysis, interpretation of data, the writing of this article or the decision to submit it for publication.
